# Supplementary material for: Using an Unsupervised Clustering Model to Detect the Early Spread of SARS-CoV-2 Worldwide
Source: Genes (Basel). 2022 Apr 7;13(4):648. doi: 10.3390/genes13040648 (PMC9030792; doi:10.3390/genes13040648)
Supplement: Supplementary file 1 [file genes-13-00648-s001.zip › Supplementary information.pdf]

# Using an Unsupervised Clustering Model to Detect the Early Spread of SARS-CoV-2 Worldwide

**Yawei Li [1], Qingyun Liu [2], Zexian Zeng [3] and Yuan Luo [1,*]**

[1] Department of Preventive Medicine, Feinberg School of Medicine, Northwestern University, Chicago, IL 60611, USA; yawei.li@northwestern.edu
[2] Department of Immunology and Infectious Diseases, Harvard T.H. Chan School of Public Health, Boston, MA 02115, USA; qingyunliu@hsph.harvard.edu
[3] Department of Data Science, Dana Farber Cancer Institute, Harvard T.H. Chan School of Public Health, Boston, MA 02215, USA; zexian_zeng@dfci.harvard.edu
* Correspondence: yuan.luo@northwestern.edu

## Supplementary Figures

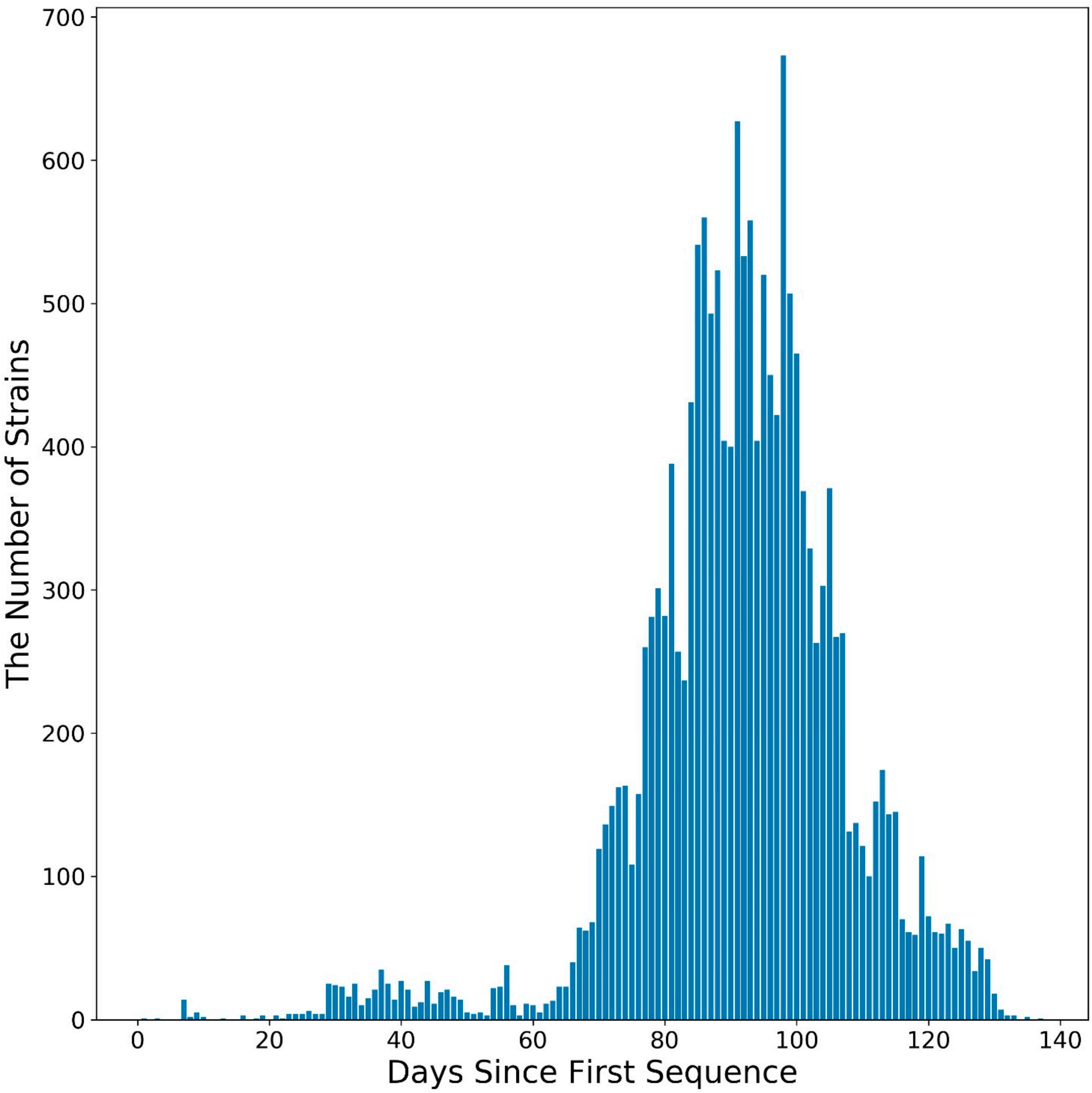


**Figure S1.** The distribution of the number of collected SARS-CoV-2 strains per day.

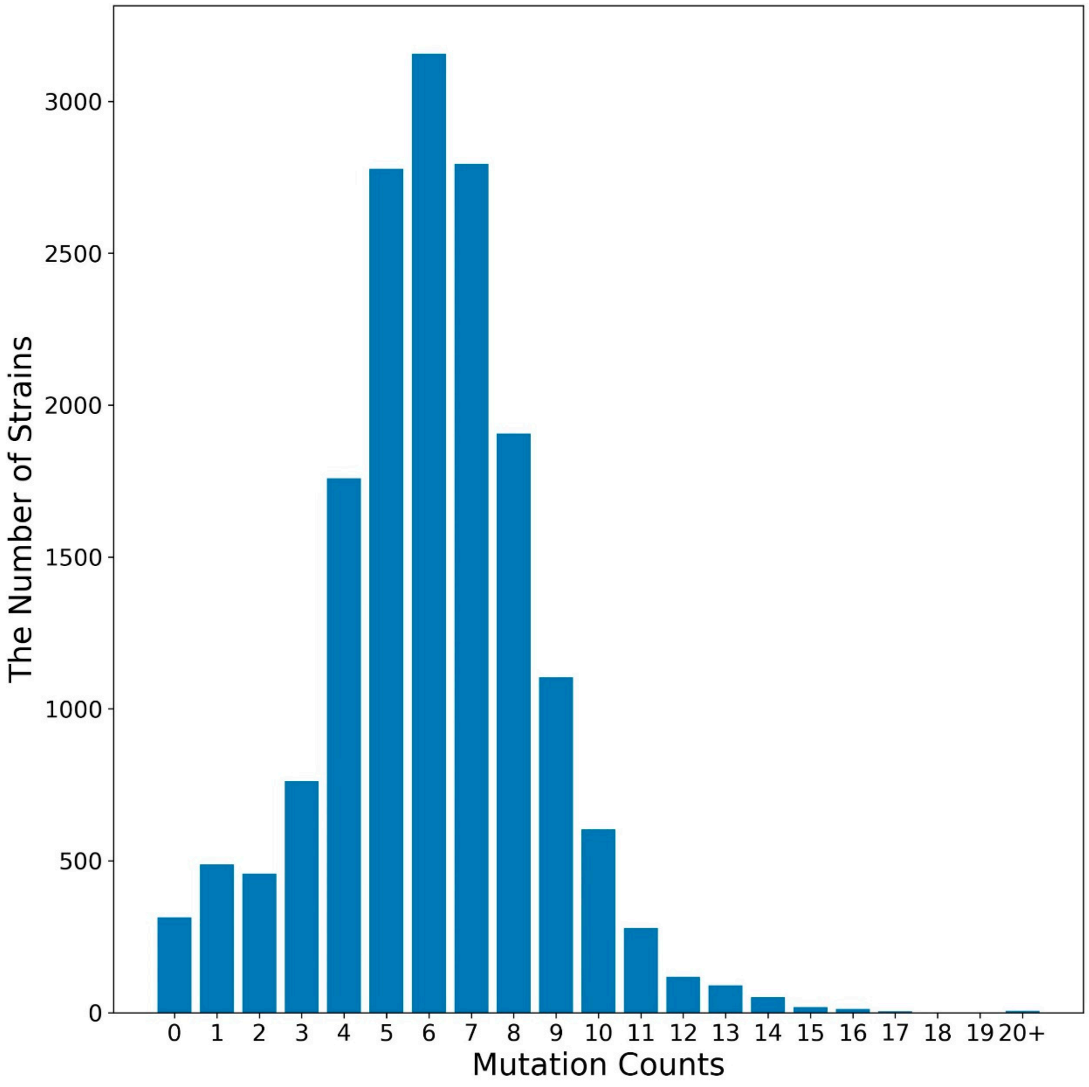


**Figure S2.** The distribution of the mutation counts of the 16,873 SARS-CoV-2 strains.

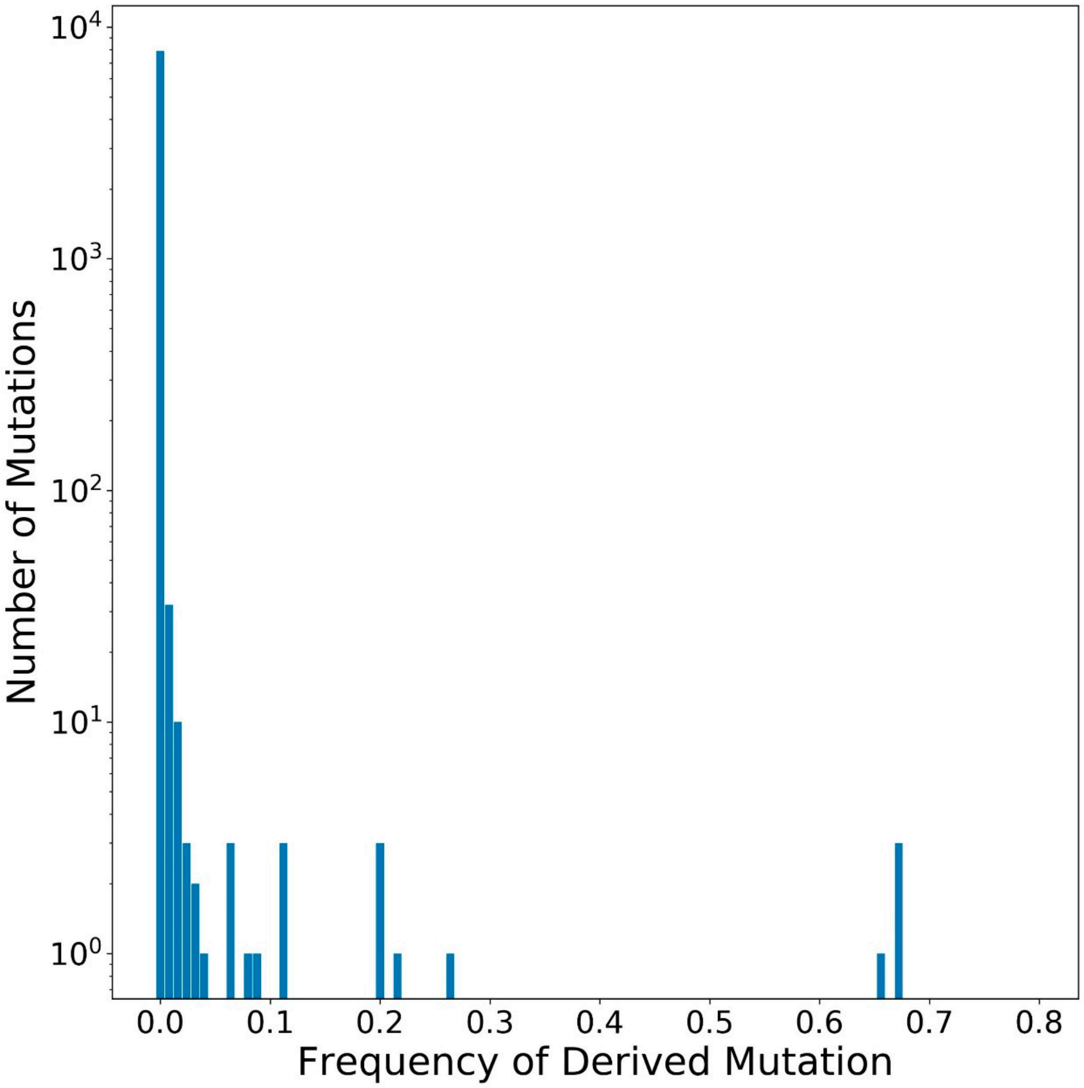


**Figure S3.** Frequency spectra of SARS-CoV-2. A log-10 scale is used for the Y axis of the graph, and the Y axis ranges from 1 to 10,000.

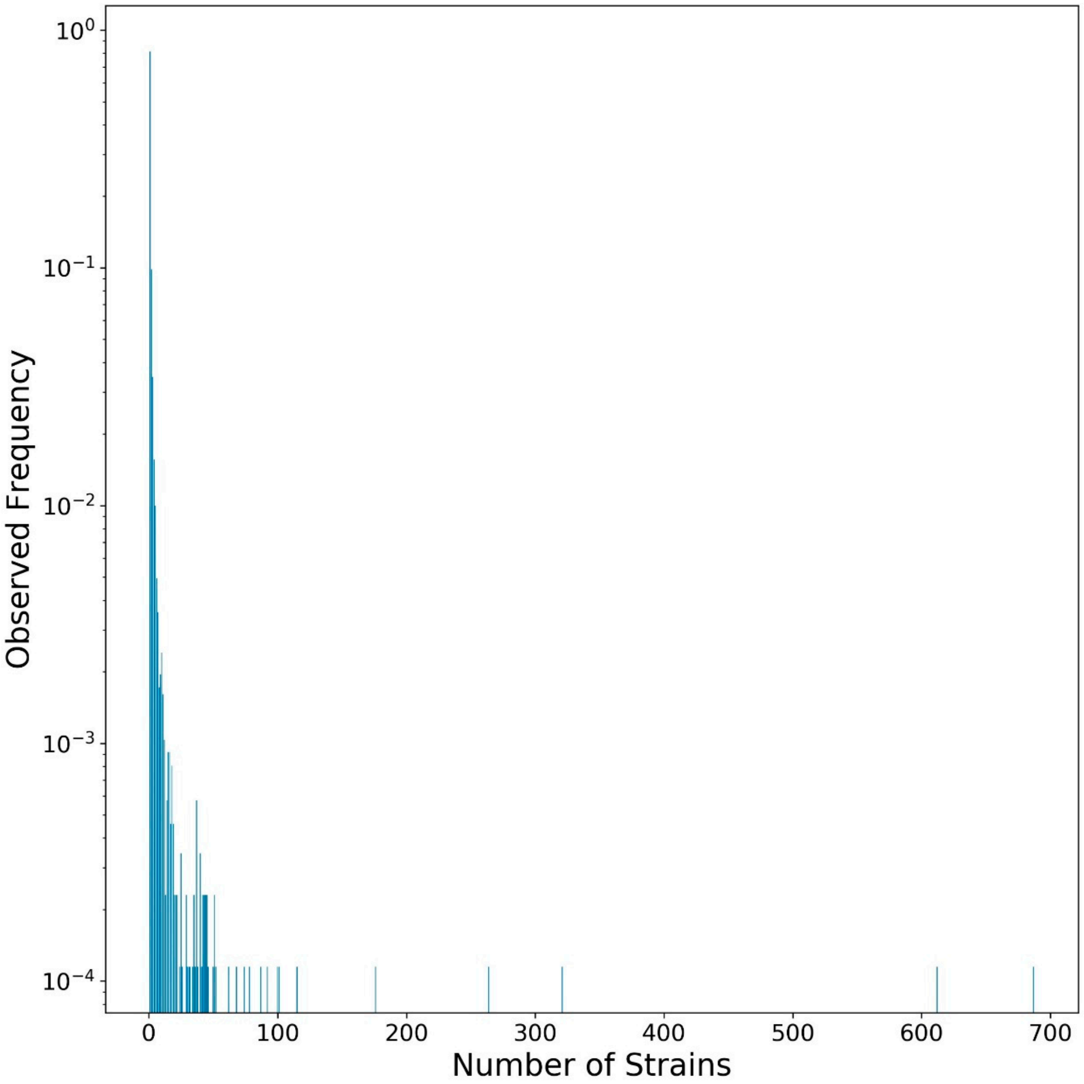


**Figure S4.** Normalized allele frequency of 16,873 SARS-CoV-2 strains. The X axis is the number of strains for each unique genome and the Y axis is the proportion of the unique genomes. A log-10 scale is used for the Y axis of the graph, and the Y axis ranges from 0.0001 to 1.

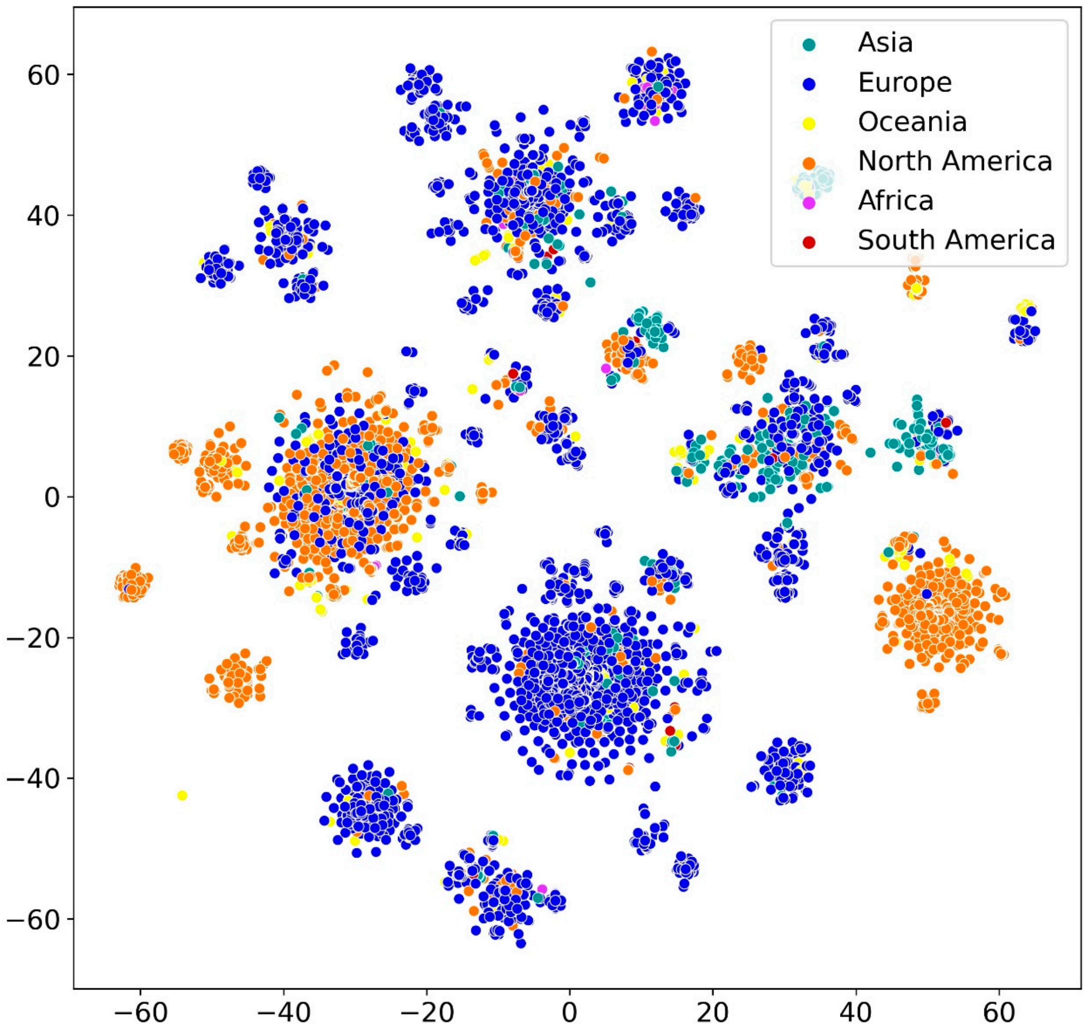


**Figure S5.** The t-SNE plot by geographic groups.

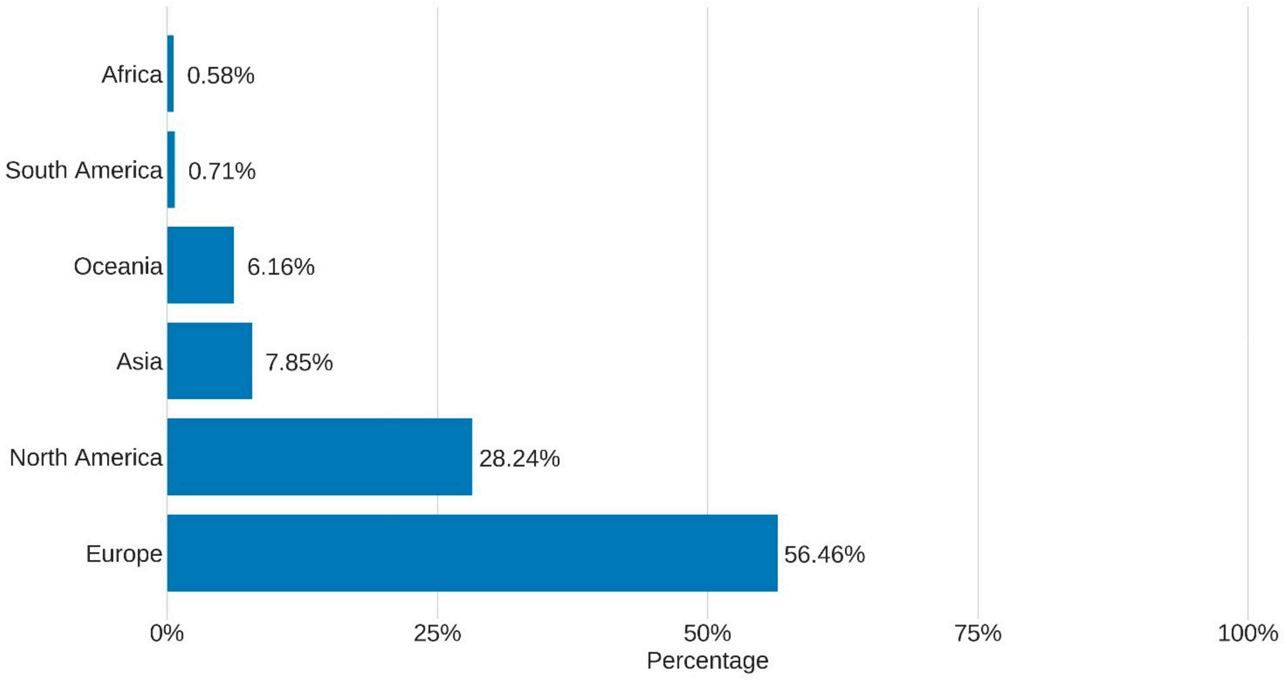


**Figure S6.** The bar chart showing the proportions of collected genomes from the six continents in the entire SARS-CoV-2 population.

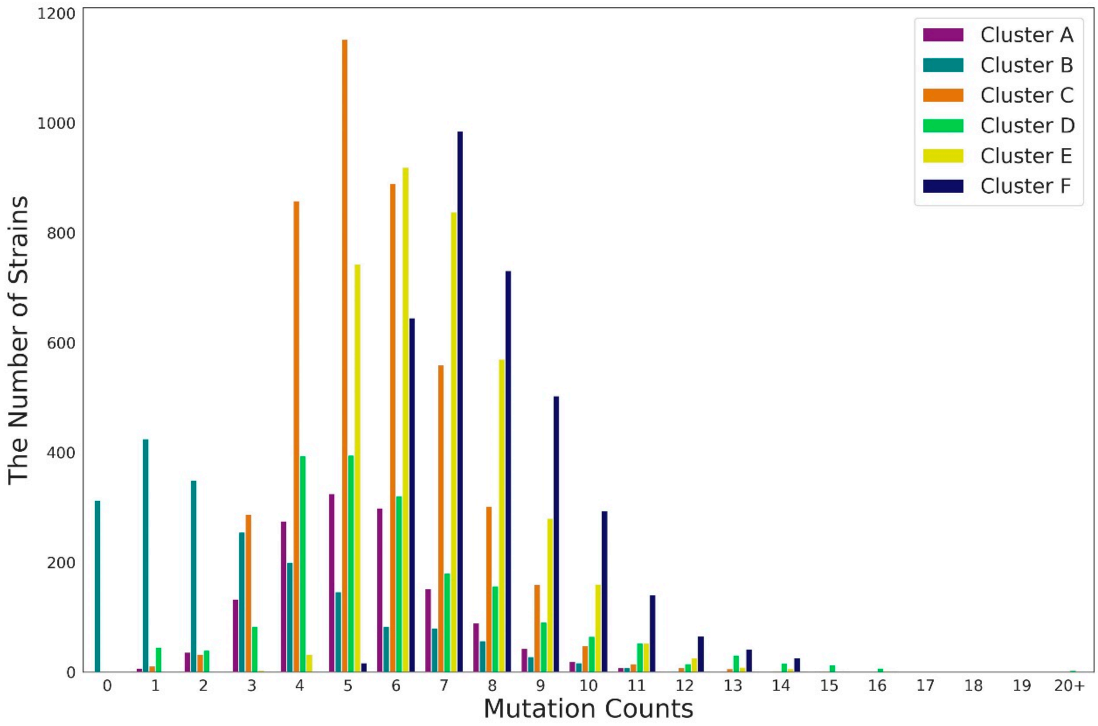


**Figure S7.** The distribution of the mutation counts of the strains for the six clusters.

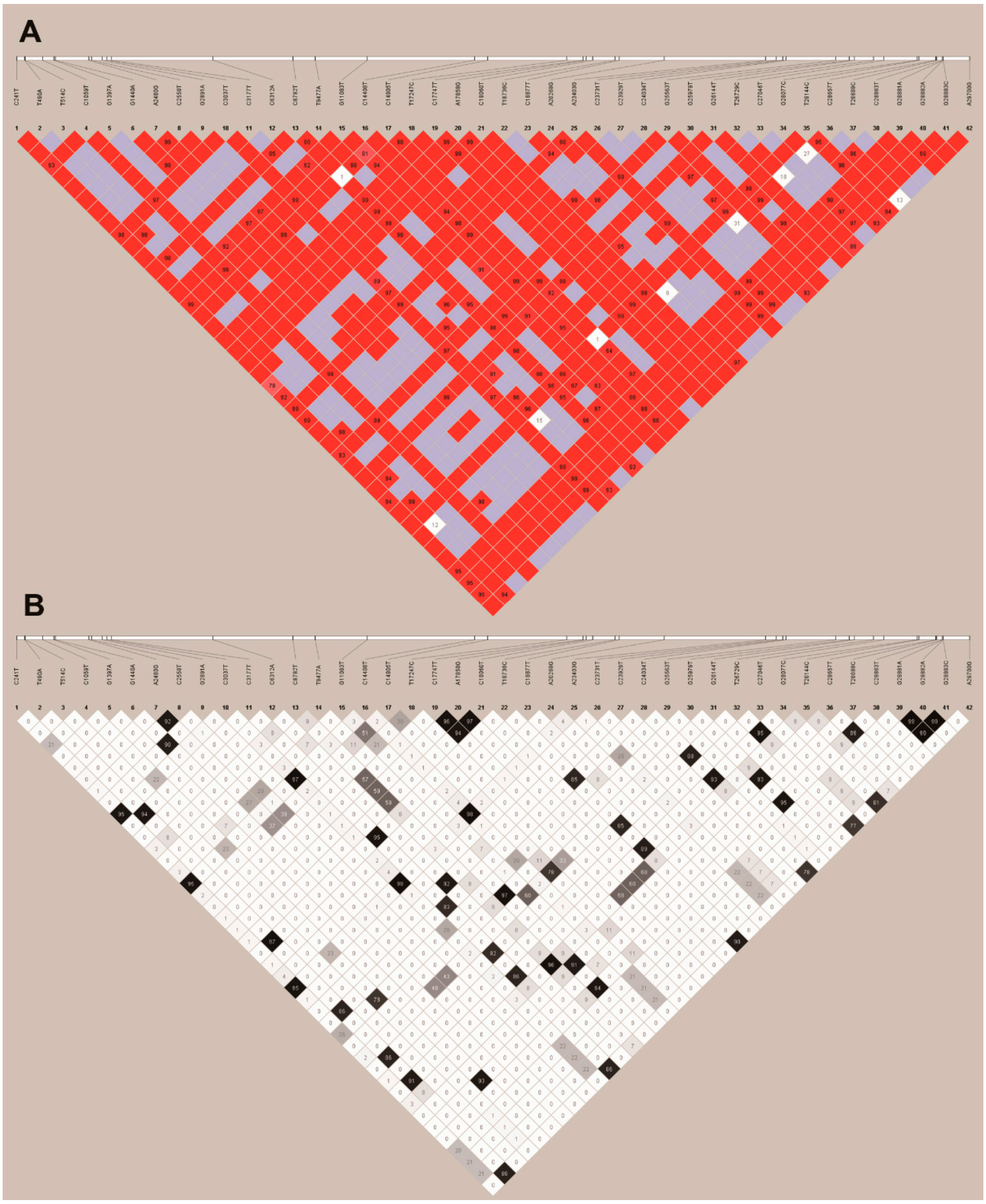


**Figure S8.** The D′ and $r^2$ of the 42 mutations. (**A**) D′ values that correspond to substitution pairs are expressed as percentages and are shown within the respective squares. Higher D′ values are indicated with a brighter red color. (**B**) The numbers within the squares represent the $r^2$ scores for pairwise LD. $r^2$ values are represented by white for $r^2 = 0$, with intermediate values for $0 < r^2 < 1$ indicated by shades of grey.

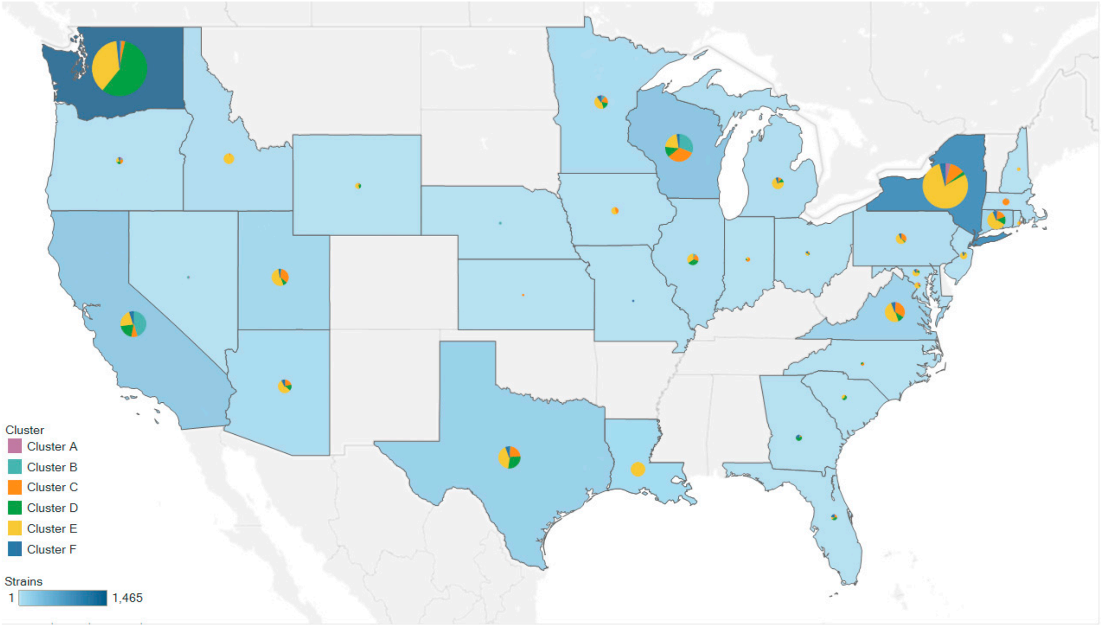


**Figure S9.** Geographic distribution of six clusters in the United States. Pie charts display the proportions of six clusters among all SARS-CoV-2 strains in each state. Circle sizes and the color scales correspond to the number of strains analyzed per state.

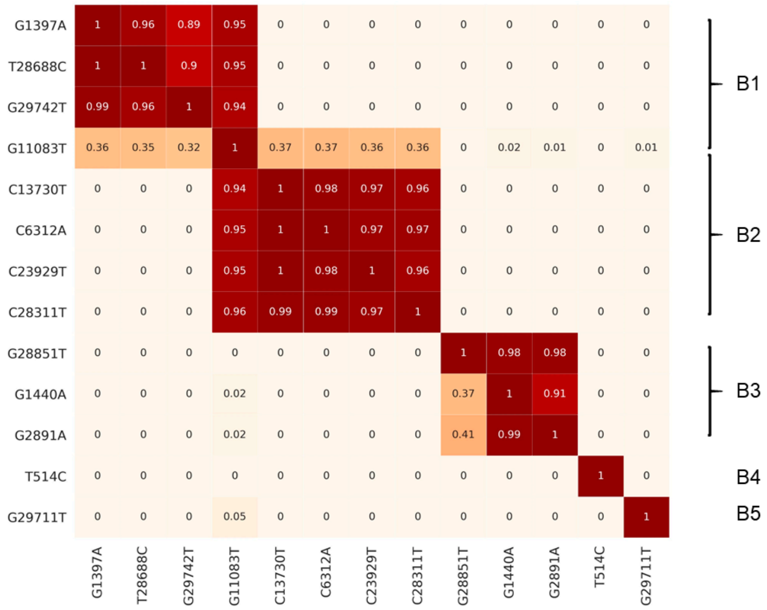

**Figure S10.** The pairwise dependency score (see Materials and Methods) of the mutations with frequency >0.05 within cluster B. The heatmap shows that there are five major subclusters within cluster B.

**Table S1:** Accession IDs with demographic information for the strains in this study.

**Table S2:** the ANOVA result for all nucleotide substitutions.
